# A Pseudo Hydronium Solvate Ionic Liquid and Taxonomy of the Allied Protic Media

**DOI:** 10.1002/asia.202500146

**Published:** 2025-06-24

**Authors:** Atsushi Kitada, Kio Kawata, Kazuhiro Fukami, Kuniaki Murase

**Affiliations:** ^1^ Department of Chemical System Engineering The University of Tokyo Hongo 7‐3‐1, Bunkyo Tokyo 113–8656 Japan; ^2^ Department of Materials Science and Engineering Kyoto University Yoshida‐hommachi 36‐1, Sakyo Kyoto 606–8501 Japan

**Keywords:** Camphorsulfonate, Crownether, p*K*_a_, Proton conduction, Pseudo hydronium solvate ionic liquid

## Abstract

A ternary equimolar mixture of water, 18‐crown‐6‐ether (18C6), and (+)‐10‐camphorsulfonic acid (CSA), [H_2_O]/[18C6]/[CSA] = 1/1/1 by mole, has been prepared and characterized as a “pseudo hydronium solvate ionic liquid (IL).” In this system, 18C6‐coordinated water is only partially protonated by CSA, resulting in a coexistence of 18C6‐solvated hydronium (H_3_O^+^) ions and neutral [H_2_O·18C6] complexes. Unlike previously reported protic media formed with superacids, which undergo complete proton transfer to form solvate ILs, this system retains a significant fraction of neutral species due to the moderate Δp*K*_a_ between CSA and water. Spectroscopic measurements including diffusion coefficient assessments reveal that proton transport proceeds via a Grotthuss‐like mechanism involving neutral complexes, distinguishing this medium from both vehicle‐type and fast‐proton‐diffusive solvate ILs. Analysis using dimensionless parameters—self‐diffusion coefficient and molar conductivity ratios—offers a quantitative framework for profiling ionicity and transport behavior in such systems. These findings provide fundamental insights into proton conduction mechanisms in pseudo‐ionic environments and establish a rational basis for designing new proton‐conducting materials. The chiral nature of the CSA^−^ anion further expands the potential of this system for applications in asymmetric catalysis, electrochemical sensing, and photoelectrochemical energy conversion devices.

## Graphical abstract

A ternary mixture of water, 18‐crown‐6‐ether (18C6), and (+)‐10‐camphorsulfonic acid (CSA) forms a “pseudo hydronium solvate ionic liquid” where water is partially protonated by CSA. The difference in p*K*_a_ between acid and base affects the proton conduction behavior, which varies across different protic ionic liquids. This behavior is mapped in a 2D plot for designing new protic media.

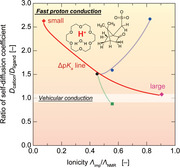

## 1 Introduction

Understanding how new properties emerge from molecular complexity is a central theme across chemistry, physics, and biology—an idea encapsulated in the phrase “More is different”.^[^ ^1^ ^]^ When multiple components are combined, interactions such as ionization, dissociation, protonation, solvation and complexation can give rise to properties not observed in the individual components. Among these, acid‐base reactions can produce ionic liquids (ILs), which have attracted considerable attentions as unconventional media in chemical, biochemical, and/or electrochemical systems.^[^ ^2^ ^]^

**Figure 1:**
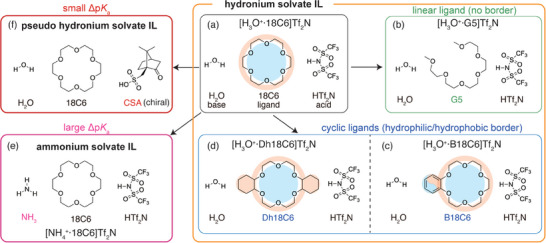
Examples of protic solvate ILs and their design principles based on Δp*K*_a_ and ligand geometry. (a) [H_3_O^+^·18C6]Tf_2_N, the first reported hydronium solvate IL,^[^ ^4a^ ^]^ (b) [H_3_O^+^·G5]Tf_2_N,^[^ ^5^ ^]^ (c) [H_3_O^+^·B18C6]Tf_2_N,^[^ ^5^ ^]^ (d) [H_3_O^+^·Dh18C6]Tf_2_N,^[^ ^5^ ^]^ (e) [NH_4_^+^·18C6]Tf_2_N, an ammonium solvate IL,^[^ ^6^ ^]^ and (f) a pseudo hydronium solvate IL composed of H_2_O, 18C6, and CSA (this work). The cyan and orange highlights over cyclic ligands in (a,c,d) represent hydrophilic and hydrophobic regions, respectively.

ILs are commonly categorized into aprotic, protic, solvate, and inorganic types.^[^ ^3^ ^]^ Solvate ILs are unique in that metal cations are chelated or coordinated by neutral ligands, giving rise to IL‐like behavior. Recently, however, a new subclass has emerged at the intersection of protic and solvate ILs: hydronium solvate ILs.^[^ ^4^ ^]^ The first such example was [H_3_O^+^·18C6]Tf_2_N (see Figure 1a), formed by mixing water (H_2_O), ligand (18‐crown‐6‐ether; 18C6), and imide superacid (HTf_2_N; Tf = CF_3_SO_2_) in a 1:1:1 molar ratio. In this system, proton transfer generates a hydronium (H_3_O^+^) ion that is stabilized by solvation with 18C6, yielding a proton‐conductive medium with relatively high ionic strength. This compound not only exhibits the highest Hammet acidity among known ILs (*H*_0_ = −4.4),^[^ ^4a^ ^]^ but also displays anomalous fast proton conduction in the absence of free neutral molecules.^[^ ^4b^ ^]^ Since then, several structural analogues of hydronium‐based and ammonium‐based solvate ILs have been reported (Figure 1b–e).^[^ ^5^, ^6^ ^]^

In protic ILs formed from equimolar acid‐base mixtures (HA + B → [HB^+^][A^−^]), full proton transfer results in the pinning of protons on the base (B), restricting mobility to vehicular transport mechanisms.^[^ ^7^ ^]^ In contrast, “pseudo protic ILs,” which arise from incomplete proton transfer, contain free neutral molecules that mediate proton hopping via Grotthuss‐like mechanisms.^[^ ^8^ ^]^ The extent of proton transfer—and thus the distinction between “protic” and “pseudo protic” ILs—is primarily governed by the difference in p*K*_a_ between the acid/base pairs, that is, Δp*K*_a_ = p*K*_a_(BH⁺) – p*K*_a_(HA).^[^ ^9^ ^]^ This thermodynamic parameter reflects the driving force for proton transfer: larger Δp*K*_a_ values favor complete proton transfer and ionic character, whereas smaller values lead to equilibrium mixtures of ionic and neutral species. Notably, although such behavior emerges spontaneously under equimolar mixing conditions, the degree of proton transfer—and thus the resulting ionicity—can be deliberately modulated by tuning Δp*K*_a_ through rational selection of acid, base, and any coordinating ligands. This suggests a framework for designing new “pseudo protic solvate ILs” by systematically tuning Δp*K*_a_ through the choice of acid, base, and ligand.

Motivated by this, we investigate a ternary equimolar mixture of H_2_O, 18C6, and (+)‐10‐camphorsulfonic acid (CSA) (Figure 1f), which forms a new type of pseudo hydronium solvate IL. Compared to HTf_2_N superacid, CAS is a weaker (yet still strong) acid (p*K*_a_ = 1.2), resulting in a smaller Δp*K*_a_ when paired with the H_3_O^+^/H_2_O couple. This system allows us to explore the impact of moderate proton donation and solvation on ionicity and transport. We characterize its physicochemical properties and compare them with other known protic solvate ILs, using dimensionless numbers that capture essential features of ionic transport.

## 2 Results and Discussions

### 2.1 [18C6]/[CSA]/[H_2_O] = 1/1/1 is Characterized as a Pseudo Hydronium Solvate IL

The obtained sample with a molar ratio of [18C6]/[CSA]/[H_2_O] = 1/1/1 was a colorless liquid at room temperature, and quantitative analyses guarantee the stoichiometry. The hydrogen, carbon, and sulfur contents are listed in Table 1, where experimental and calculated values show good agreement, although the experimental carbon content was 0.6 wt% lower than the calculated value. The Karl‐Fischer titration revealed a water content of 3.56 wt%, in good agreement with the ideal value of 3.50 wt%.

**Table 1:** Results of elemental analysis for [18C6]:[CSA]:[H_2_O] = 1/1/1.

Element	H (wt%)	C (wt%)	S (wt%)
Experimental	8.24	50.76	6.19
Calculated	8.23	51.35	6.23

**Figure 2:**
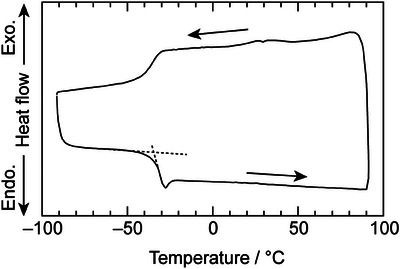
DSC curves of [18C6]/[CSA]/[H_2_O] = 1/1/1.

In the differential scanning calorimetry (DSC) curves (Figure 2), no peaks corresponding to pure 18C6, water, and CSA were observed, indicating that the ternary equimolar mixture did not show the physicochemical properties of the neutral raw materials, similar to the hydronium solvate ILs.^[^ ^4^, ^5^ ^]^ However, the mixture did not show any crystallization behavior, but instead showed a unique glass transition at −34 °C. This is in stark contrast to the analogous [H_3_O^+^·18C6] salts (Tf_2_N^−^, ClO_4_^−^, SbF_6_^−^, PF_6_^−^, TfO^−^, BF_4_^−^, FeCl_4_^−^) where clear melting points were observed.^[^ ^4^, ^10^ ^]^ It may be considered a superior feature that the system remains liquid at room temperature, as related compounds using 18C6 tend to melt at somewhat higher temperatures.

**Figure 3:**
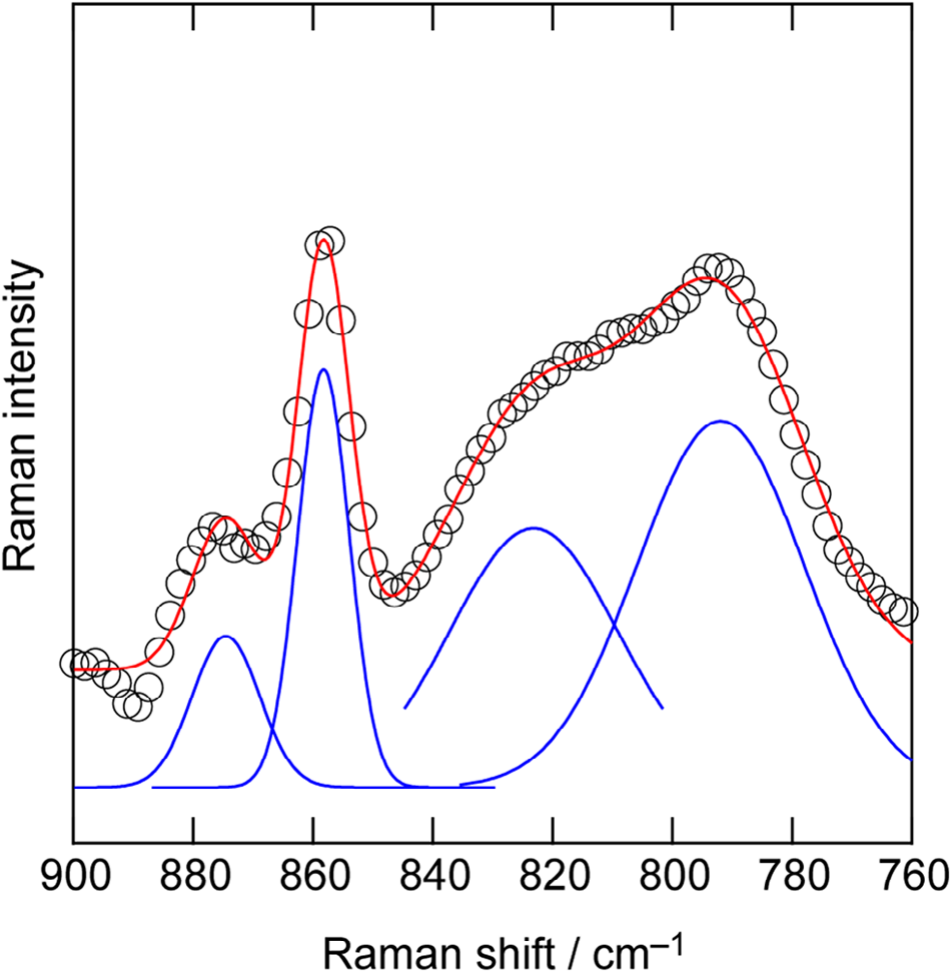
Raman spectra for [18C6]/[CSA]/[H_2_O] = 1/1/1 at room temperature (open circles) in the range of 760–900 cm^−1^ and the Gaussian fitting results (blue and red curves).

Figure 3 shows the Raman spectra for the [18C6]/[CSA]/[H_2_O] = 1/1/1 mixture. The band centered at 875 cm^−1^ originates from the ─COC─ stretching vibration and CH_2_ rocking vibration of [H_3_O^+^·18C6].^[^ ^4a^ ^]^ A band at lower wavenumber, centered at 858 cm^−1^, may correspond to another conformation of [H_3_O^+^·18C6] with weaker coordination. According to ab initio calculations, the bands for [H_3_O^+^·18C6] appear as a B_1_ mode at 860 cm^−1^, and as B_2_, B_3_, and B_4_ modes in the low wavenumber region (828, 810, and 803 cm^−1^) with an area ratio of 22:1:3:6.^[^ ^11^ ^]^ The calculated band positions align well with the deconvolution results of the experimental spectra for [18C6]/[CSA]/[H_2_O] = 1/1/1, similar to those observed in hydronium solvate IL.^[^ ^4a^ ^]^ Therefore, it can be concluded that the experimental spectra show the B_1_ mode, with major intensity, corresponding to the bands centered at 875 and 858 cm^−1^, while the B_2_–B_4_ modes overlap to compose the broad bands in the lower wavenumber region.

In Figure 3, however, the resulting area ratio for the experiments, estimated through Gaussian fitting, is 1:3.3, which is significantly larger than the area ratio of 1:0.54 (= 22:(1 + 3 + 6)).^[^ ^11^ ^]^ Additionally, similar broad bands are observed for acid‐free samples, viz. pure 18C6, [18C6]/[H_2_O] = 1/1 and [18C6]/[H_2_O] = 1/2 (Figure S1). Therefore, the excess area intensity in the low wavenumber region suggests a reduced amount of H_3_O^+^ or the presence of neutral H_2_O, that is, unprotonated [18C6·H_2_O] in the CSA system.

**Figure 4:**
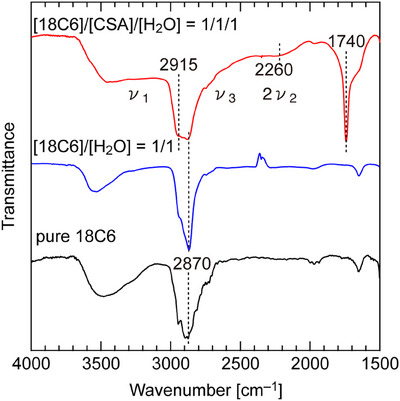
FT‐IR spectra between 4000–1500 cm^−1^ for [18C6]/[CSA]/[H_2_O] = 1/1/1, [18C6]/[H_2_O] = 1/1, and pure 18C6.

Figure 4 represents the FT‐IR spectra for [18C6]/[CSA]/[H_2_O] = 1/1/1, [18C6]/[H_2_O] = 1/1, and pure 18C6. Absorption bands characteristic of H_3_O^+^ appear at wavenumbers of 2780–3250 cm^−1^ (ν_1_), 1048–1182 cm^−1^ (ν_2_), 2500–3100 cm^−1^ (ν_3_), and 1477–1705 cm^−1^ (ν_4_), and the band centered at 2260 cm^−1^ may be attributed to the first overtone (2ν_2_) of H_3_O^+^ symmetric rocking vibration ν_2_.^[^ ^4^, ^10^ ^]^ The spectra for [18C6]/[CSA]/[H_2_O] = 1/1/1 shows a distinct absorption centered at 2915 cm^−1^ in addition to 2870 cm^−1^ characteristic to the acid‐free samples, which may result from the conformational changes of 18C6 by the encapsulation of H_3_O^+^. The observation of the two kinds of 18C6 C─H stretching vibration proves the coexistence of H_3_O^+^ and H_2_O in [18C6]/[CSA]/[H_2_O] = 1/1/1, in agreement with the Raman results.

Consequently, the ternary equimolar mixture [18C6]/[CSA]/[H_2_O] = 1/1/1, where the protonation of the H_2_O is imperfect, do not satisfy the criteria of hydronium solvate ILs, which requires no free neutral molecules be present.^[^ ^4a^ ^]^ Compared to the superacid HTf_2_N (p*K*_a_ ≈ −4),^[^ ^9a^ ^]^ CSA, while still a strong acid, is notably weaker (p*K*_a_ = 1.2). As a result, its combination with weakly basic H_2_O under the same 18C6 coordination environment leads to a smaller Δp*K*_a_. The CSA protic media can be categorized as a “pseudo protic solvate IL” in contrast to the Tf_2_N^−^ counterpart with full protonation, yet solvated hydronium ions are partially present. Hence, we prefer to name it a “pseudo hydronium solvate ionic liquid”, where the protonation of H_2_O is imperfect, leading to volatile nature and the prohibited crystallization or the glass transition.

## 3 Protonation State in the Pseudo Hydronium Solvate Ionic Liquid

**Figure 5:**
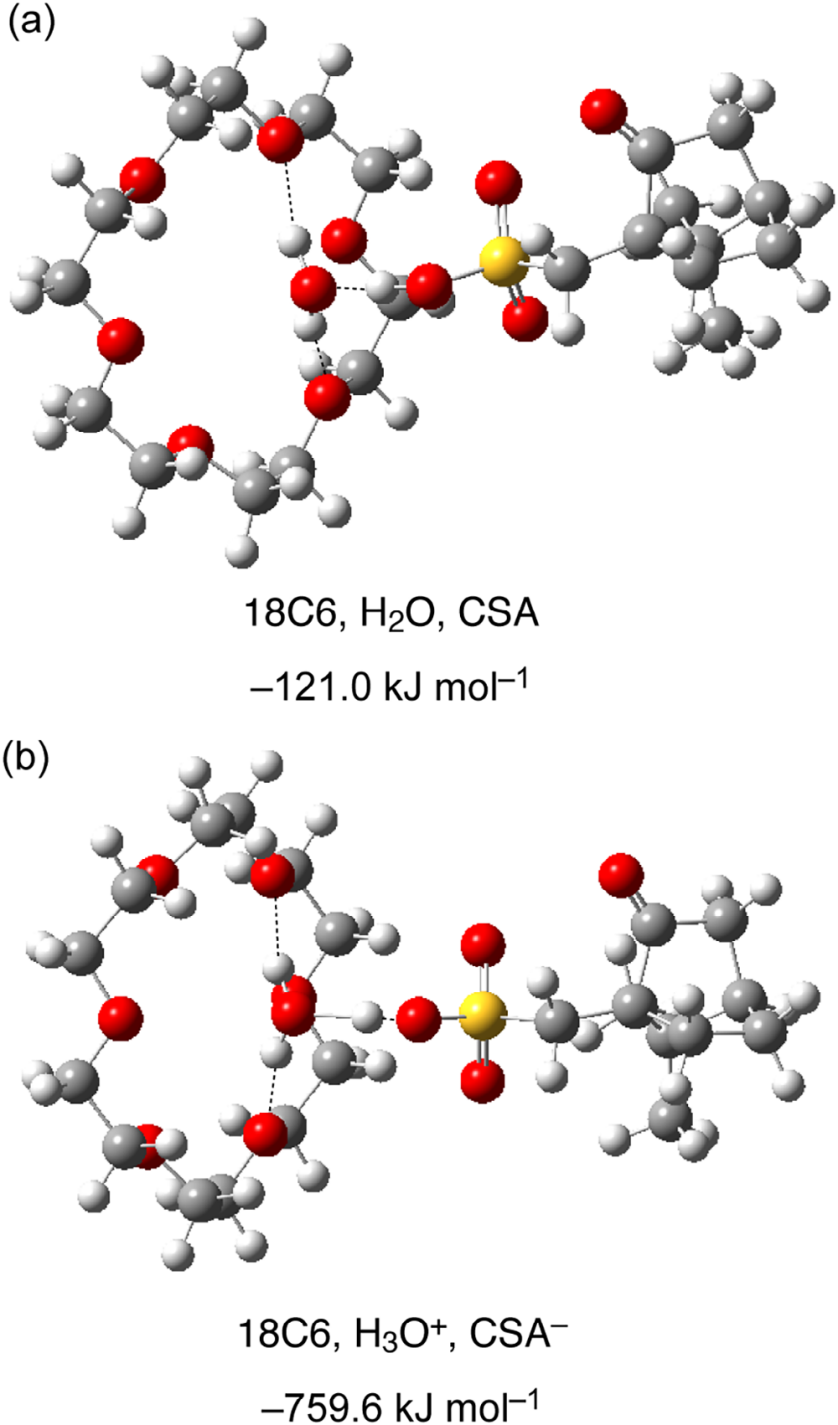
Optimized structures of the system containing (a) 18C6, CSA, and H_2_O and (b) 18C6, CSA^−^, and H_3_O^+^. white: H, gray: C, red: O, yellow: S.

Figure 5 shows the optimized structures based on ab initio molecular orbital calculations (see Supporting Information for details) and the corresponding formation energies of two systems: one composed of neutral molecules (18C6, CSA, and H_2_O), and the other composed of ionic species (18C6, CSA^−^ anion, and H_3_O^+^ cation). The ionic system exhibits much lower energies for the ionic system, attributed to the Coulombic interactions. However, in both systems, the two O atoms of 18C6 coordinate to the two H atoms of H_2_O or H_3_O^+^. More importantly, H_3_O^+^ is coordinated not only by 18C6 but also by the CSA^−^ anion, in stark contrast to the Tf_2_N^−^ counterpart system, where all three protons of H_3_O^+^ are bound to the oxygens of 18C6.^[^ ^6^ ^]^ Therefore, [H_3_O^+^·18C6] and CSA^−^ can be considered partially associated in the pseudo hydronium solvate IL. In other words, the competition between 18C6 ligands and the conjugate CSA^−^ anions governs the equilibria of H_3_O^+^.

**Figure 6:**
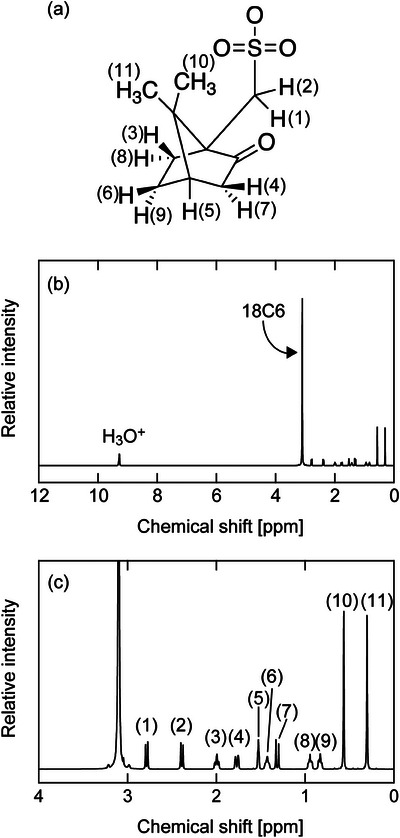
(a) Chemical structure of CSA with assignment numbers, (b) ^1^H NMR spectrum of the [18C6]/[CSA]/[H_2_O] = 1/1/1 mixture at 75 °C, and (c) its enlarged view.

Figure 6 presents the ^1^H NMR spectrum of the pseudo hydronium solvate IL, along with the assignment of each signal. The signal for H_3_O^+^ appears at a chemical shift of 9.28 ppm, the signal for 18C6 at 3.10 ppm, and those for CSA^−^ in the higher magnetic field region (0–3 ppm). The integral intensity ratio of H_3_O^+^, 18C6 (C_12_H_24_O_6_), and CSA^−^ (C_10_H_16_O_4_S) is 1:8:5.3, confirming the stoichiometry of [18C6]/[CSA]/[H_2_O] = 1/1/1 for the timescale of the NMR measurements. More importantly, since the signal corresponding to H_3_O^+^ in the hydronium solvate IL [H_3_O^+^·18C6]Tf_2_N appears at a lower magnetic field 10.85 ppm,^[^ ^4a^ ^]^ the acidity of the [18C6]/[CSA]/[H_2_O] = 1/1/1 is weaker, and the degree of dissociation is lower, which is consistent with the fact that the raw material CSA is less acidic than HTf_2_N. Additionally, for 18C6, the signal in [18C6]/[CSA]/[H_2_O] = 1/1/1 is distinct from those of pure 18C6 (3.26 ppm), [H_3_O^+^·18C6]Tf_2_N (3.23 ppm),^[^ ^4a^ ^]^ and [18C6]/[H_2_O] = 1/1 (3.22 ppm) (Figure S2a), further confirming the imperfect protonation and/or encapsulation.

The ^13^C NMR data, along with signal assignments, are shown in Figure S3. The signal for 18C6 appears at 69.84 ppm in the [18C6]/[CSA]/[H_2_O] = 1/1/1, with all other signals attributed to CSA^−^. Pure 18C6 shows a signal at 70.43 ppm,^[^ ^4a^ ^]^ and [18C6]/[H_2_O] = 1/1 at 70.14 ppm (Figure S2b), both of which are distinct from the pseudo hydronium solvate IL. These results support the conclusion drawn from the ^1^H NMR measurements.

While Δp*K*_a_ provides a thermodynamic descriptor for the degree of proton transfer in equimolar acid‐base systems, it is also informative to consider how this relates to the actual pH of the medium. In aqueous systems, pH is defined by the activity of free hydronium ions, but in complex media such as (pseudo) protic ILs, the concept of pH becomes less straightforward due to the low dissociation degree.^[^ ^9^ ^]^ Nevertheless, a smaller Δp*K*_a_, as in the case of CSA versus water compared to HTf_2_N versus water, implies a less complete proton transfer. This partial protonation is reflected in the coexistence of [H_2_O·18C6] and [H_3_O⁺·18C6] species, and leads to a medium where the effective pH is buffered and less acidic than that of fully ionic hydronium solvate ILs (e.g., *H*_0_ = −4.4^[^ ^4a^ ^]^). Such behavior emphasizes the nuanced interplay between Δp*K*_a_ and measurable acidity, reinforcing the classification of the present system as a pseudo hydronium solvate IL.

## 4 Proton Conduction Properties of the Pseudo Hydronium Solvate IL

**Figure 7:**
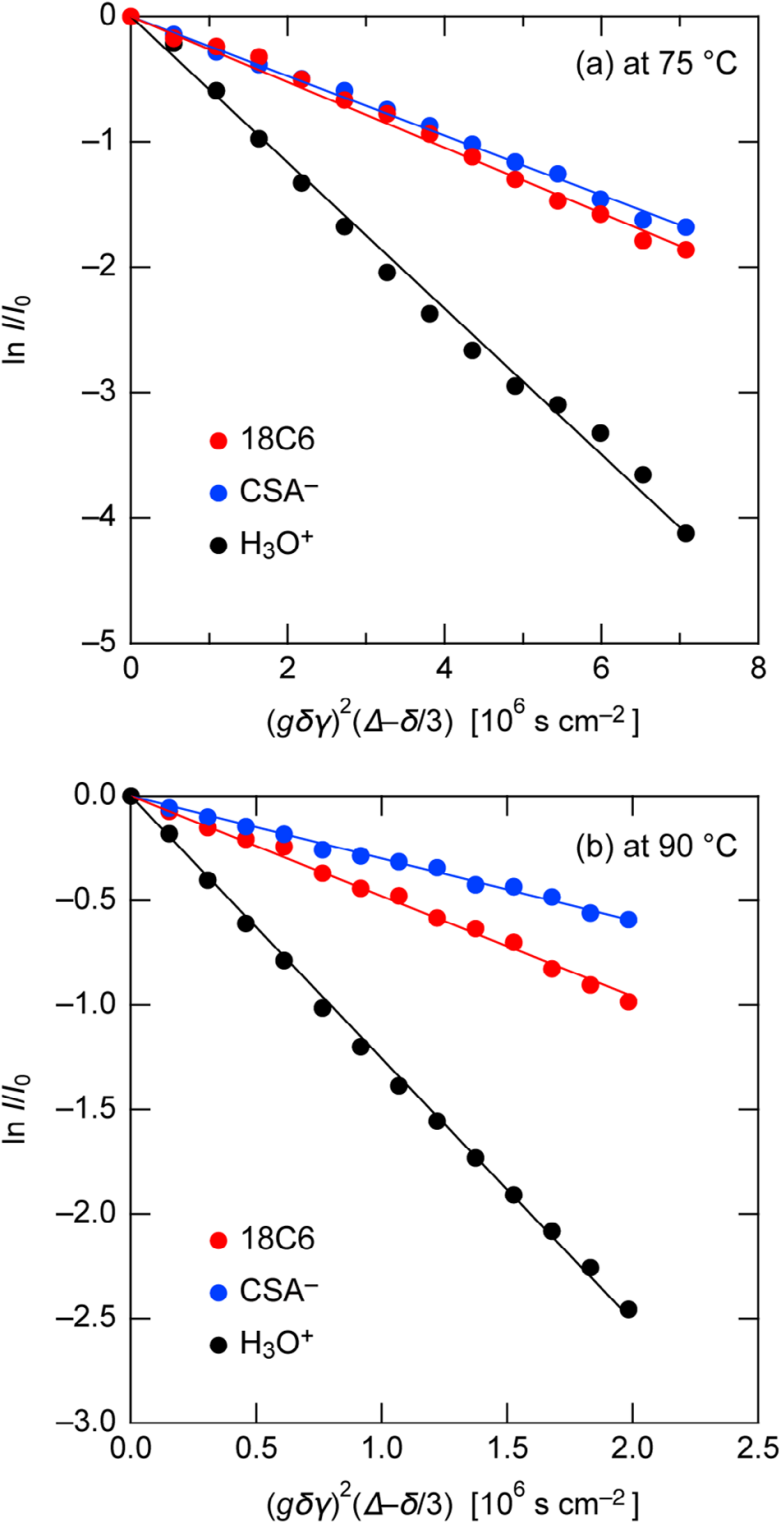
Plots of echo signal decay for 18C6 (red), CSA^−^ (blue), and H_3_O^+^ (black) in the [18C6]/[CSA]/[H_2_O] = 1/1/1 mixture at (a) 75 °C and (b) 90 °C based on the Stejskal equation. *R* ^2^ values for linear fits are as follows: at 75 °C, 0.99 (18C6), 0.98 (CSA^−^), and 0.99 (H_3_O^+^); at 90 °C, 0.99 (18C6), 0.98 (CSA^−^), and 0.99 (H_3_O^+^). Fitting errors are within ±3%.

The experimental realization of pseudo protic solvate IL provides a platform to systematically investigate how moderate proton donation and specific solvation environments influence the resulting ionicity and proton transport behavior. Figure 7 shows the plot of the ^1^H echo signal decay based on Stejskal's equation,^[^ ^12^ ^]^ which exhibits a linear relationship. To aid in evaluating model accuracy, *R* ^2^ values and fitting errors for each plot are provided in the caption. Table 2 lists the self‐diffusion coefficients (*D*) for the 18C6 ligand (*D*_18C6_), CSA^−^ anion (*D*_CSA__−_), and the proton of H_3_O^+^ (*D*_H3O__+_), all determined from the slope of the plot.

**Table 2:** Self‐diffusion coefficients (10^−7^ cm^2^ s^−1^) of 18C6, CSA^−^, and H_3_O^+^ in the [18C6]/[CSA]/[H_2_O] = 1/1/1 mixture at 75 °C and 90 °C.

Temperature (°C)	*D*_18C6_	*D*_CSA_^−^	*D*_H3O_^+^	*D*_H3O_^+^/*D*_18C6_
75	2.61	2.37	5.83	2.2
90	4.79	2.98	12.6	2.6

At both 75 °C and 90 °C, *D*_H3O+_ was found to be larger than the other diffusion coefficients, indicating that the hydronium‐derived protons diffuse faster than 18C6 in the pseudo hydronium solvate IL. This observation is consistent with previous studies on structurally related Tf_2_N‐based hydronium solvate IL, which also report fast proton diffusion (*D*_H3O+_ > *D*_18C6_), validating our results.^[^ ^4b^ ^]^ In contrast to other types of conventional solvate ILs, where proton motion is primarily governed by vehicular transport,^[^ ^4^, ^5^, ^6^, ^7^, ^8^ ^]^ the CSA‐based pseudo hydronium solvate IL likely facilitates proton motion via a Grotthuss‐ike proton jump mechanism involving neutral species.^[^ ^7^ ^]^ This is attributable to the partial dissociation and the existence of neutral [H_2_O·18C6] complexes.

**Figure 8:**
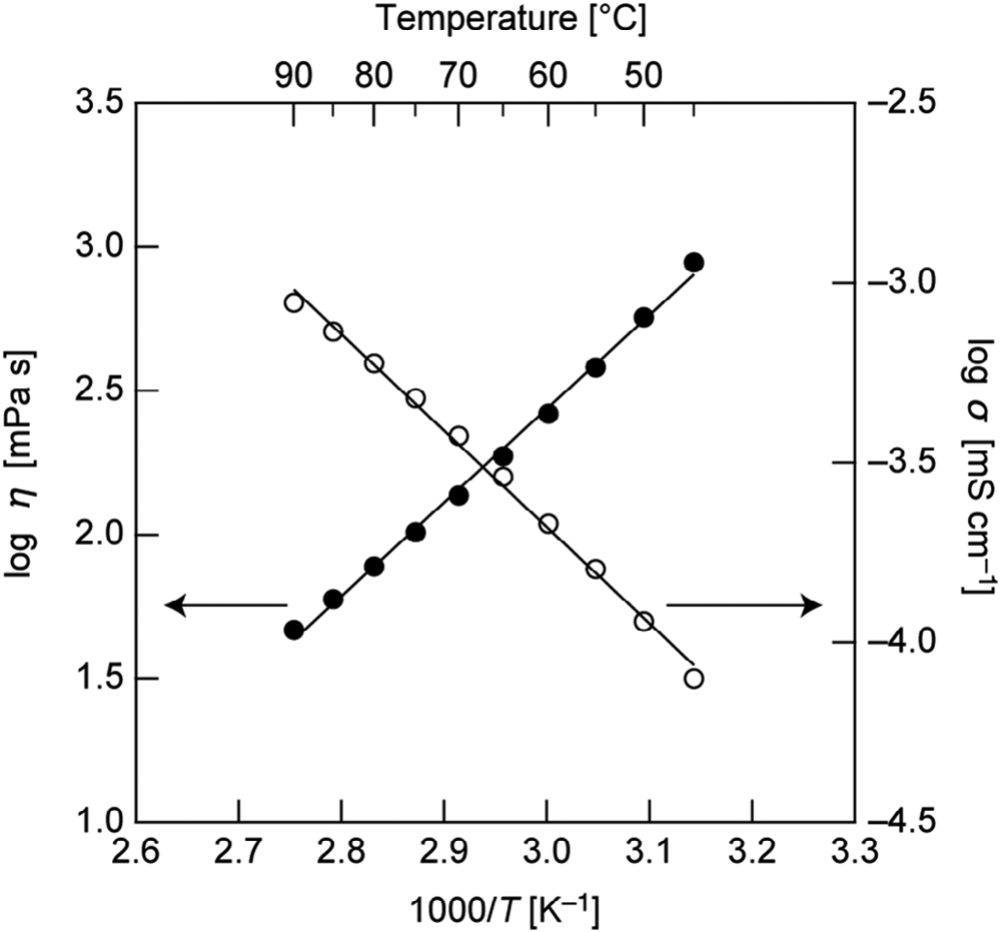
Arrhenius plots for the conductivities and viscosities of [18C6]/[CSA]/[H_2_O] = 1/1/1.

The temperature dependence of conductivity and viscosity (Arrhenius plots) for the [18C6]/[CSA]/[H_2_O] = 1/1/1 mixture between 45 °C and 90 °C is shown in Figure 8, with the values of conductivity and viscosity summarized in Table S1. It is evident that the activation energy for conductivity (51.3 kJ mol^−1^) is approximately 10 kJ mol^−1^ lower than that for viscosity (62.3 kJ mol^−1^), strongly suggesting the involvement of a proton jump mechanism.

The absolute value of ionic conductivity in the liquid state is relatively low compared to other systems, remaining below 1 mS cm^−1^ even at 90 °C. This is lower than those of hydronium/ammonium solvate ionic liquids, as well as pseudo‐protic ionic liquids such as the CH_3_COOH–imidazole system.^[^ ^4^, ^5^, ^6^, ^7^, ^8^ ^]^ In pseudo protic ILs, the concentration of ionic species is estimated to be less than 1%—a level undetectable by Raman spectroscopy—yet ionic conductivity arises through Grotthuss‐like hopping of a very small number of protons.^[^ ^7^ ^]^ In contrast, the pseudo hydronium solvate IL studied here contains a detectable amount of ionic species—more than an order of magnitude higher—yet still exhibits lower conductivity and higher viscosity. In this sense, the pseudo solvate IL occupies an intermediate position bridging pseudo protic ILs and conventional protic solvate ILs.

To assess the contribution of self‐diffusion (*Λ*_NMR_) obtained by PGSE‐NMR to the actual ion conduction (*Λ*_imp_) measured by electrochemical impedance, ionicity is defined as the molar conductivity ratio, *Λ*_imp_/*Λ*_NMR_.^[^ ^4^, ^5^, ^8^ ^]^ The molar conductivity *Λ*_NMR_ is given by the Nernst‐Einstein equation *Λ*_NMR_ = *F* ^2^(*D*_cation_ + *D*_anion_)/*RT*​ where *F* is the Faraday constant, *R* is the gas constant, and *T* is the absolute temperature. In this context, ion‐ion interactions, which influence the actual conduction in ILs, are not accounted for in *Λ*_NMR_; instead, independent ionic migration is assumed. The ionicity values for the [18C6]/[CSA]/[H_2_O] = 1/1/1 mixture at 75 °C and 90 °C were calculated to be 0.073 and 0.074, respectively. These values are well below the typical threshold of 0.4, which is commonly used to identify solvate ionic liquids. Consequently, similar to the conclusions drawn from static characterization, this system cannot be classified as a conventional solvate IL.^[^ ^13^ ^]^

## 5 Mapping the CSA System in the Protic Solvate IL Regime

In the above context, the presence of neutral water species in the equimolar ternary mixture [H_2_O]/[18C6]/[CSA] = 1/1/1 suggests that the system does not meet the strict criteria of a conventional solvate IL. However, this composition represents a valuable example of a pseudo protic solvate IL, lying at the boundary between molecular and ionic regimes. As such, it serves as an insightful comparison point with fully ionic protic solvate ILs. Below, we demonstrate how our findings contribute to the broader understanding of the relationship between Δp*K*_a_ and the properties of protic solvate ILs.

**Figure 9:**
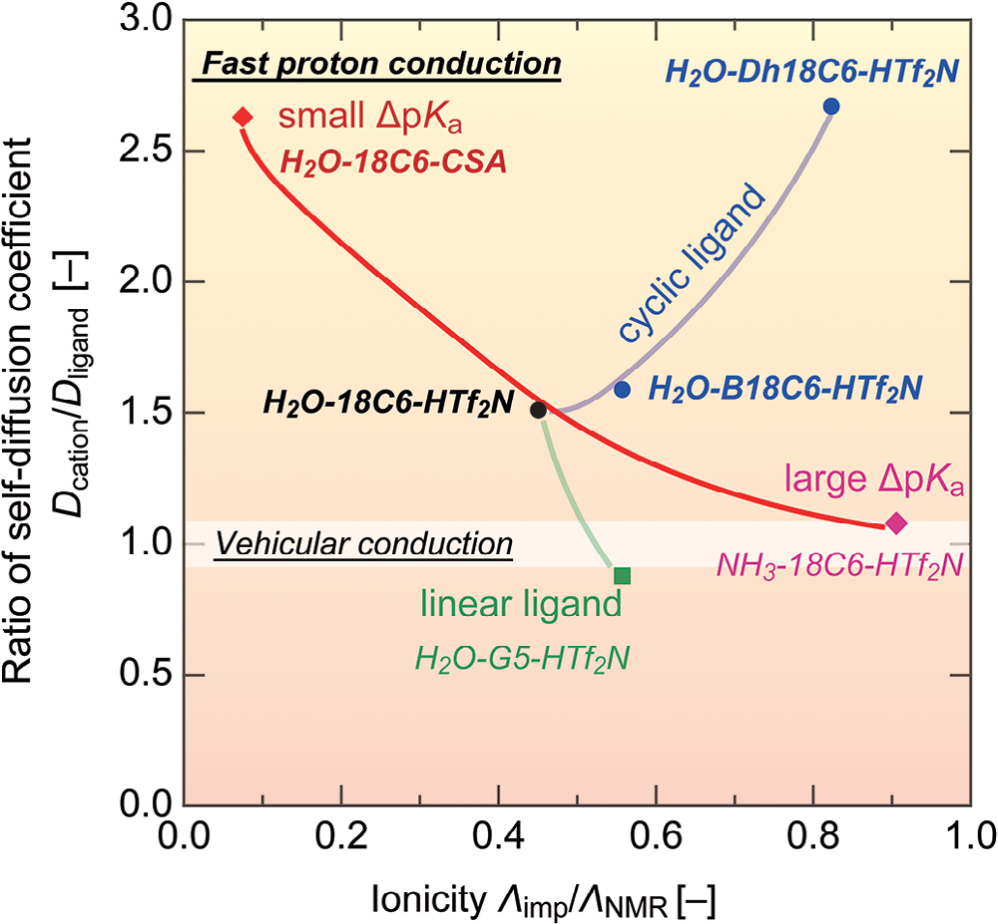
Plots of *D*_cation_/*D*_ligand_ versus ionicity (*Λ*_imp_/*Λ*_NMR_) for the protic solvate ionic liquids including pseudo‐protic ionic liquid H_2_O‐18C6‐CSA.

Figure 9 illustrates the relationship between the self‐diffusion coefficient ratio (*D*_cation_/*D*_ligand_) and the ionicity (*Λ*_imp_/*Λ*_NMR_) for the base:ligand:acid = 1:1:1 system, referred to as base‐ligand‐acid in the figure. A white line, where *D*_cation_/*D*_ligand_ = 1 (effectively in the ratio of 0.9–1.1), is drawn to indicate vehicular conduction. The criterion for fast proton conduction is *D*_cation_/*D*_ligand_ > 1.1.^[^ ^4^, ^5^, ^6^ ^]^

First, the ligand is fixed as 18C6, and the effect of Δp*K*_a_ is investigated (red lines in Figure 9). Specifically, compared to [H_3_O^+^·18C6]Tf_2_N (black circle), Δp*K*_a_ decreases when the anion is changed from Tf_2_N^−^ to CSA^−^ anion, whereas Δp*K*_a_ increases when the cation is changed from H_3_O^+^ to NH_4_^+^. In the H_2_O‐18C6‐CSA system, *Λ*_imp_/*Λ*_NMR_ decreases by an order of magnitude (*Λ*_imp_/*Λ*_NMR_ = 0.07) compared to [H_3_O^+^·18C6]Tf_2_N (0.45), indicating that the contribution of ionic diffusion to actual conduction is low, and the system is not highly “ionic”. Thus, the obtained mixture can be defined as a pseudo hydronium solvate IL, similar to a pseudo “protic” IL, for example, an equimolar mixture of imidazole and acetic acid, where proton transfer is imperfect.^[^ ^8^ ^]^ Because neutral molecules are present in the H_2_O‐18C6‐CSA system, the value of *D*_cation_/*D*_ligand_ (= 2.6) is significantly larger than that for [H_3_O^+^·18C6]Tf_2_N (*D*_cation_/*D*_ligand_ = 1.5).^[^ ^4b^ ^]^ Notably, since CSA contains a chiral carbon, [H_2_O]/[18C6]/[CSA] = 1/1/1 forms a chiral pseudo protic IL. This system may provide an interesting medium for not only asymmetric organic synthesis,^[^ ^14^ ^]^ but also energy applications, as CSA derivatives have recently been used in dye sensitized‐ and perovskite solar cells, and protic IL is applied to nonaqueous proton batteries.^[^ ^15^ ^]^When the cation is changed from H_3_O^+^ to NH_4_^+^ to reduce the acidity, an ammonium solvate IL, [NH_4_^+^·18C6]Tf_2_N (Figure 1e), is obtained.^[^ ^6^ ^]^ Compared to the strongly acidic compound [H_3_O^+^·18C6]Tf_2_N,^[^ ^4a^ ^]^ Δp*K*_a_ increases, and *Λ*_imp_/*Λ*_NMR_ also rises to 0.91, while *D*_cation_/*D*_ligand_ decreases to 1.1. In other words, the substitution of H_3_O^+^ with NH_4_^+^ shifts the conduction mechanism from fast proton conduction to vehicular conduction. This vehiclular conduction strongly suggests a long residence time of 18C6 with the NH_4_^+^ cation. Consequently, the thermal stability of the IL is enhanced, with stability up to 200 °C. The conductivity at 150 °C reaches 10 mS cm^−1^, which is one order of magnitude higher than that of the [H_3_O^+^·18C6]Tf_2_N hydronium solvate IL.^[^ ^4a^ ^]^

Next, we examine the effect of ligand shape on proton conduction. In contrast to the 18C6 counterpart, replacing the ligand with a chain polyether (pentaethylene glycol dimethyl ether; G5; Figure 1b), suppresses fast proton conduction, and the vehicular conduction mechanism is observed for the chain ligands (green lines in Figure 9). Regarding ligand geometry, cyclic ligands are distinctly divided into hydrophilic and hydrophobic regions, located inside and outside the ring, respectively (see Figure 1b–d; cyan and orange region, respectively). In contrast, this distinction is not observed for linear ligands. As suggested by ab initio calculations, cyclic ligands are more likely to adopt a liquid structure in which the hydrophilic regions are spatially contiguous, facilitating fast proton conduction.^[^ ^5^ ^]^ This hypothesis is supported by 2D mapping, where both *D*_cation_/*D*_ligand_ and *Λ*_imp_/*Λ*_NMR_ values increase with increasing hydrophobicity outside the ring (18C6 < B18C6 < Dh18C6; blue lines in Figure 9).

Finally, we note that the concept of forming proton‐conduction paths by partitioning hydrophilic and hydrophobic regions at the molecular level may be analogous to the “hydropathy” of amino acids, peptides, and proteins,^[^ ^16^ ^]^ where amphiphilic units are linked to form macroscopic pathways for water and protons. Further studies could focus on the ligand effects in CSA systems, specifically by tuning the proton‐conduction pathways within the hydrophilic regions.

## 6 Conclusions

We have prepared and characterized a ternary equimolar mixture [18C6]/[CSA]/[H_2_O] = 1/1/1 as a model system mimicking known protic solvate ionic liquids. Spectroscopic and computational analyses reveal a significant presence of neutral [H_2_O·18C6] complexes, leading us to classify this system as a pseudo hydronium solvate ionic liquid. The reduced Δp*K*_a_ and diminished ionicity (*Λ*_imp_/*Λ*_NMR_) suggest a fundamentally different mode of ionic behavior compared to conventional solvate ILs. The observed proton diffusion dynamics (*D*_H3O⁺_/*D*_ligand_ > 1) are consistent with a Grotthuss‐type hopping mechanism between neutral species. Importantly, the incorporation of the chiral CSA^−^ anion imparts asymmetry to the medium, opening avenues for applications in chiral proton‐conducting materials, such as in asymmetric catalysis, electrochemical sensing, and photoelectrochemical energy conversion systems. This study underscores the potential of pseudo‐ionic systems as a platform for supramolecular protonics. By adjusting Δp*K*_a_ values and tuning ligand architecture, a wide array of protic solvate systems with tailored ion transport properties can be designed. These findings offer fundamental insight and practical guidance for the development of next‐generation materials in energy and catalysis. Further investigation into the pseudo hydronium solvate IL is expected to yield functional properties, and additional studies—such as full molecular dynamics simulations or simulations of extended clusters—would be beneficial to verify the underlying proton conduction mechanism.

## Conflict of Interests

The authors declare no conflict of interest.

## Supporting information



Supporting Information

## Data Availability

The data that support the findings of this study are available from the corresponding author upon reasonable request.
