# Supplementary material for: A Pseudo Hydronium Solvate Ionic Liquid and Taxonomy of the Allied Protic Media
Source: Chem Asian J. 2025 Jun 24;20(18):e00146. doi: 10.1002/asia.202500146 (PMC12450038; doi:10.1002/asia.202500146)
Supplement: Supplementary file 1 — Supporting Information [file ASIA-20-e00146-s001.docx]

**Supporting information**

**Methods**

18-crown-6-ether (18C6; 99.0% purity, 264.32 g/mol) was purchased from Kanto Chemical. (+)-10-camphorsulfonic acid (CSA; >98.0% purity, 232.29 g mol^–1^) was purchased from Tokyo Chemical Industry. These chemicals were stored in an argon-filled glovebox and used for the synthesis of ILs without further purification. Ultrapure water is produced by Millipore Milli-Q. To avoid moisture exposure inside the glovebox, ultrapure water was transferred into a septum-sealed screw-cap vial, which was then sealed and brought into the glovebox. Inside the glovebox, 18-crown-6 (18C6) and an equimolar amount of camphorsulfonic acid (CSA) were weighed and placed into a separate septum-sealed screw-cap vial along with a magnetic stir bar. An equimolar amount of ultrapure water was then added to this vial using a gas-tight syringe by withdrawing it from the pre-prepared water vial. The equimolar mixture of 18C6, CSA, and H_2_O ([18C6]/[CSA]/[H_2_O] = 1/1/1) was heated and stirred in the glovebox at 80°C and 500 rpm for 6 h. For comparison, two neutral system [18C6]/[H_2_O] = 1/1 and [18C6]/[H_2_O] = 1/2 were also obtained by stirring with 500 rpm at 60 °C for 6 h.

Elemental analyses were conducted by a combustion method. Thermal properties were measured using differential scanning calorimeter (DSC, Rigaku DSC8231; 5 °C min^–1^, between –90 °C and 90 °C). For Raman spectroscopy, an integrated Raman system (B&W Tek, InnoRam 785) was used, which consist of a semiconductor laser light source (785 nm), an axial transmissive spectrograph, a holographic probe head, and a CCD detector. Infrared (IR) measurements were conducted using a JASCO FT/IR–460 plus system. For the IR measurement, Nujol and hexachlorobutadiene mulls painted on a barium fluoride window were used.

Ab initio molecular orbital calculations were performed using Gaussian 16.^[1]^ The geometry of [18C6]/[CSA]/[H_2_O] = 1/1/1 was fully optimized at the B3LYP/6-311+G** level. Instead of a vacuum- or gas-phase condition, the solvation model, namely, the SMD method, was used with tetrahydrofuran (THF) as the solvent phase under a tetrahydrofuran atmosphere, because the dielectric permittivity is similar to that of common ILs.^[2]^

One-dimensional ^1^H and ^13^C nuclear magnetic resonance (NMR) spectra were obtained for liquidous [18C6]/[CSA]/[H_2_O] = 1/1/1 and [18C6]/[H_2_O] = 1/1 at 75°C. In addition, pulse-field gradient spin echo (PGSE)-NMR (^1^H) was conducted for [18C6]/[CSA]/[H_2_O] = 1/1/1 to estimate self-diffusion coefficients of each component. The magnetic field gradient pulse width was set to *δ* = 4 ms, and the diffusion time was *Δ* = 0.1 s. The magnetic field gradient strength (*g*) was varied in the range of 0.010–0.790 T m^–1^ at 75 °C, and in the range of 0.010–0.420 T m^–1^ at 90 °C.

Ionic conductivity and viscosity measurements were conducted in the temperature range of 45–90 °C. The former was determined by electrochemical impedance spectroscopy (Bio-Logic, VSP-300) using a self-made Teflon cell with stainless steel electrodes. The cell constant was calibrated with 0.1 and 1 mol dm^–3^ KCl aqueous solutions. The measurement was performed in a thermostatic chamber (Espec, SU-222). Viscosity measurements were performed between the same temperature ranges using a viscometer (Kyoto Electronics Manufacturing, EMS-1000S). Additionally, the density at room temperature for the mixture [18C6]/[CSA]/[H₂O] = 1/1/1 was calculated from mass and volume measurements, determined to be 1.27 g cm^–3^.

**References**

[1]. M. J. Frisch, G. W. Trucks, H. B. Schlegel, G. E. Scuseria, M. A. Robb, J. R. Cheeseman, G. Scalmani, V. Barone, G. A. Petersson, H. Nakatsuji, X. Li, M. Caricato, A. V. Marenich, J. Bloino, B. G. Janesko, R. Gomperts, B. Mennucci, H. P. Hratchian, J. V. Ortiz, A. F. Izmaylov, J. L. Sonnenberg, D. Williams-Young, F. Ding, F. Lipparini, F. Egidi, J. Goings, B. Peng, A. Petrone, T. Henderson, D. Ranasinghe, V. G. Zakrzewski, J. Gao, N. Rega, G. Zheng, W. Liang, M. Hada, M. Ehara, K. Toyota, R. Fukuda, J. Hasegawa, M. Ishida, T. Nakajima, Y. Honda, O. Kitao, H. Nakai, T. Vreven, K. Throssell, J. A. Montgomery, Jr., J. E. Peralta, F. Ogliaro, M. J. Bearpark, J. J. Heyd, E. N. Brothers, K. N. Kudin, V. N. Staroverov, T. A. Keith, R. Kobayashi, J. Normand, K. Raghavachari, A. P. Rendell, J. C. Burant, S. S. Iyengar, J. Tomasi, M. Cossi, J. M. Millam, M. Klene, C. Adamo, R. Cammi, J. W. Ochterski, R. L. Martin, K. Morokuma, O. Farkas, J. B. Foresman, D. J. Fox, Gaussian 16, Revision A.03, Gaussian Inc., Wallingford CT **2016**.

[2] C. Daguenent, P. J. Dyson, I. Krossing, A. Oleinikova, J. Slattery, C. Wakai and H. Weingärtner, *J. Phys. Chem. B*, **2006**, *110*, 12682–12688.


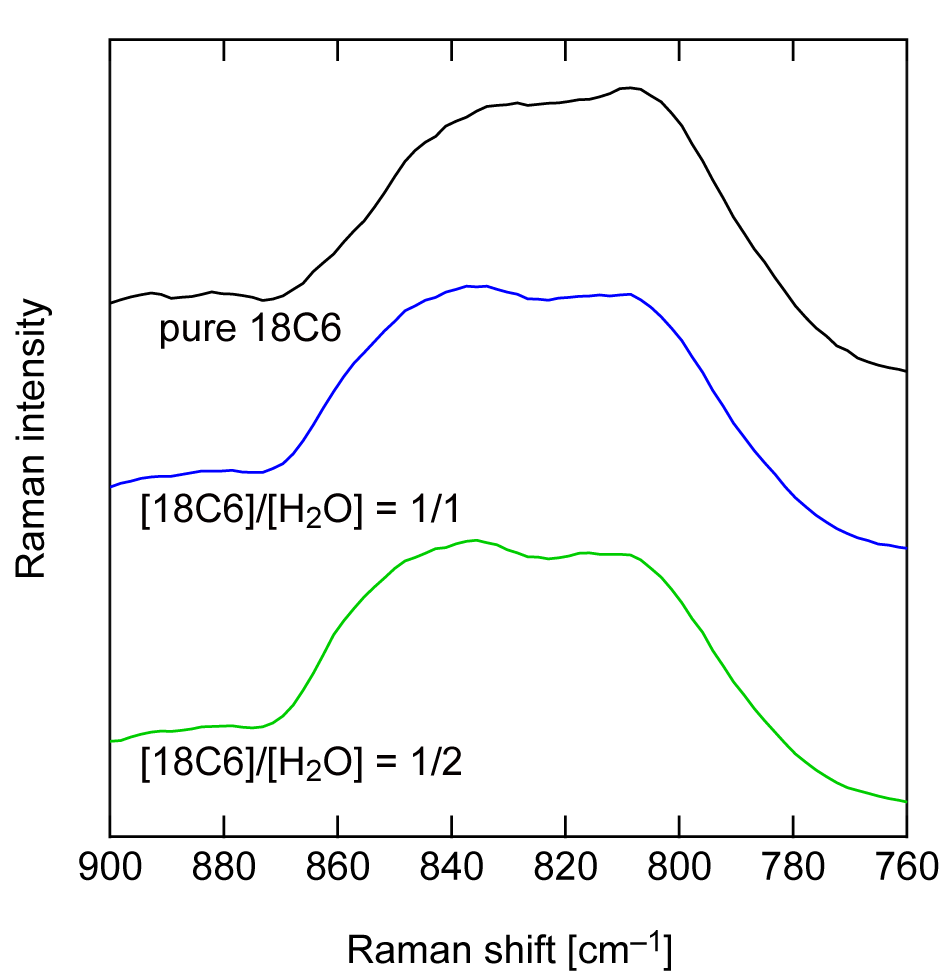


**Figure S1.** Raman spectra for (a) pure 18C6 liquid at 60 °C, (b) [18C6]/[H_2_O] = 1/1 at room temperature, and (c) [18C6]/[H_2_O] = 1/2 at room temperature.


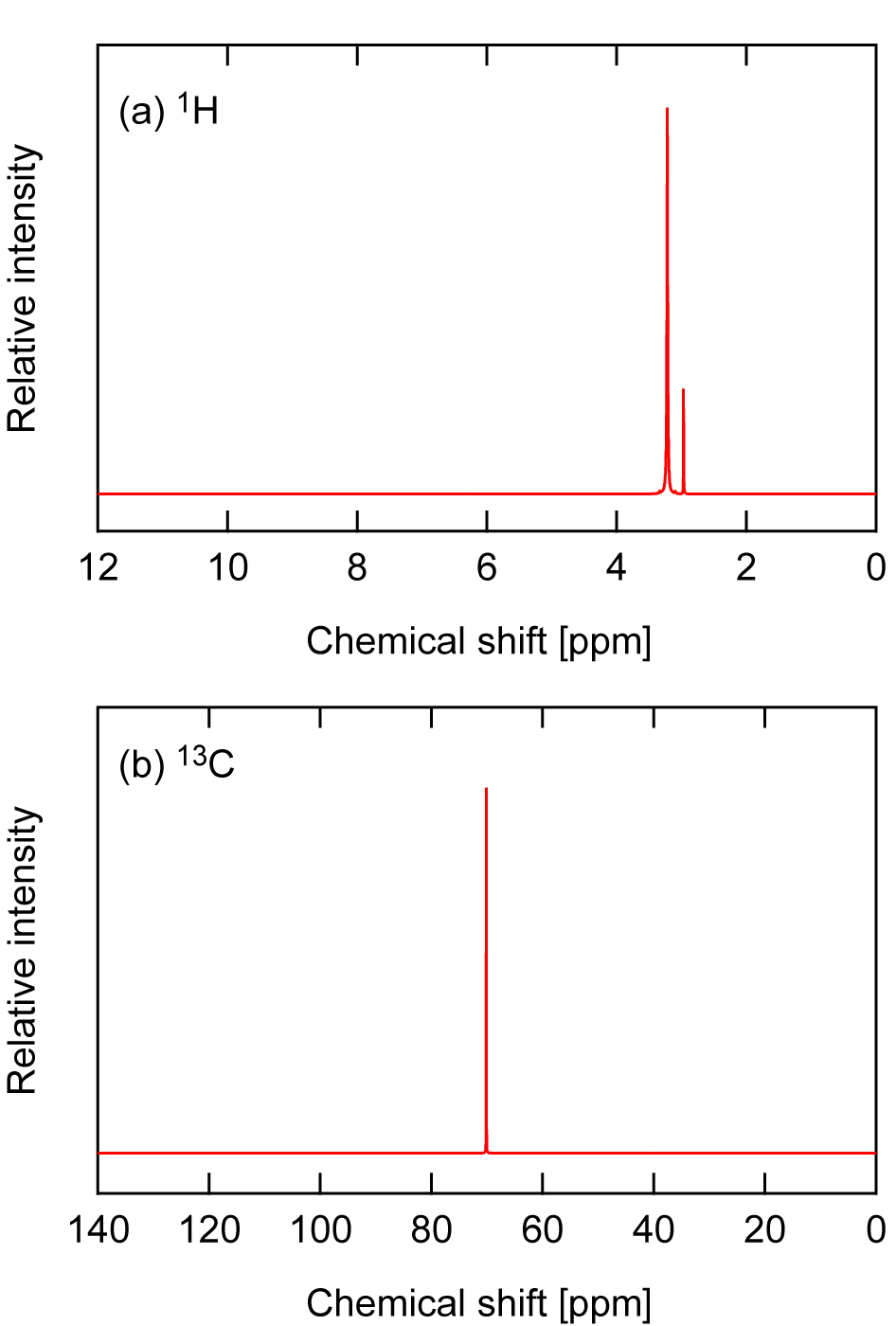


**Figure S2.** (a) ^1^H and (b) ^13^C NMR spectra for [18C6]/[H_2_O] = 1/1 at 25°C.


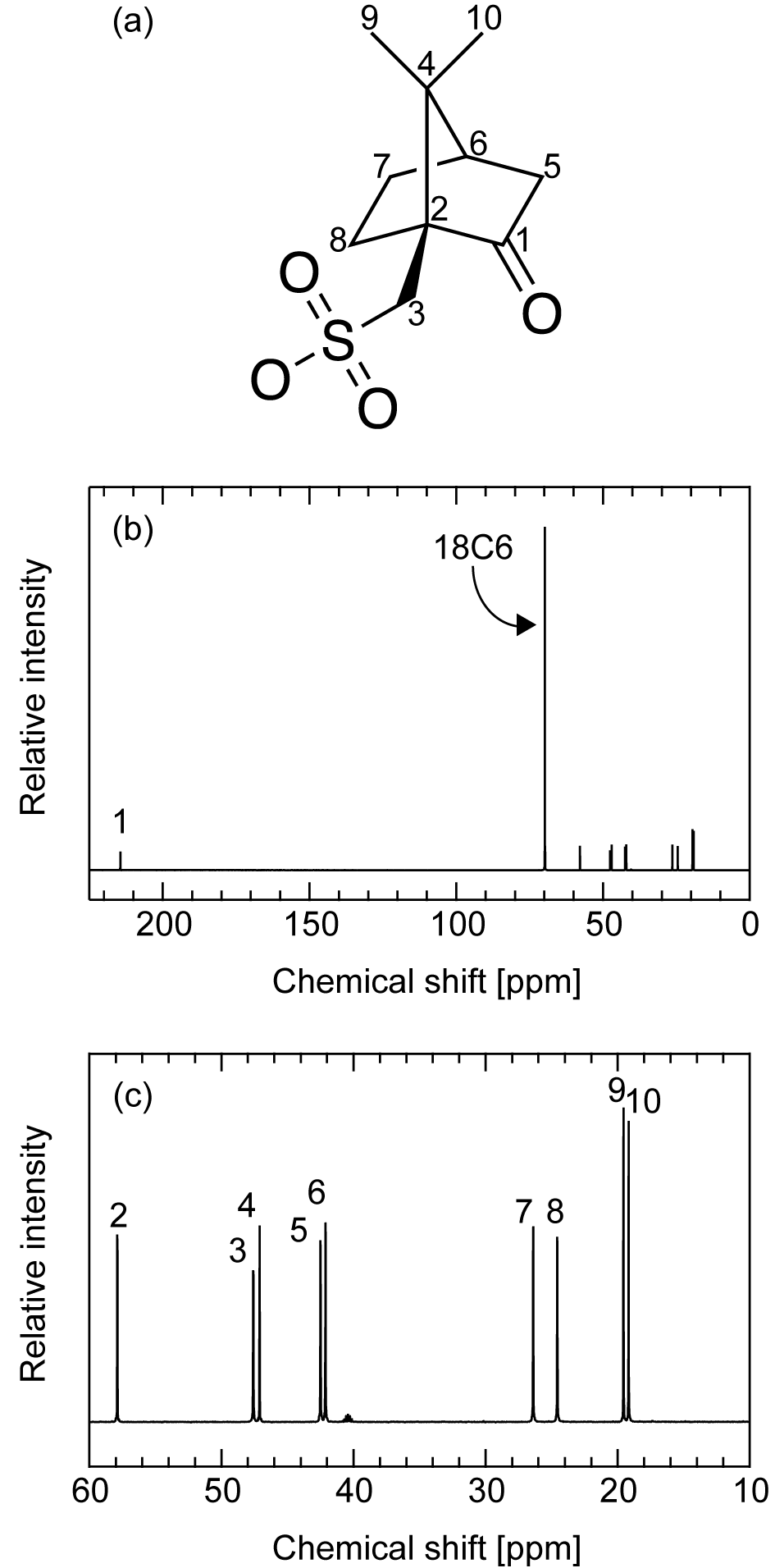


**Figure S3.** (a) Chemical structure of CSA with assignment numbers, (b) ^13^C NMR spectrum of the [18C6]/[CSA]/[H₂O] = 1/1/1 mixture at 75°C, and (c) its expanded view.

| Table S1 Physicochemical properties of [18C6]/[CSA]/[H_2_O] = 1/1/1. | | |
| --- | --- | --- |
| Temperature / °C | Ionic conductivity / mS cm^–1^ | Viscosity / mPa s |
| 45 | 0.079 | 884 |
| 50 | 0.11 | 570 |
| 55 | 0.16 | 382 |
| 60 | 0.21 | 263 |
| 65 | 0.29 | 187 |
| 70 | 0.38 | 137 |
| 75 | 0.48 | 102 |
| 80 | 0.60 | 77.5 |
| 85 | 0.73 | 59.8 |
| 90 | 0.88 | 46.7 |
